# Biological markers and cardiac remodelling following the myocardial infarction

**DOI:** 10.18632/aging.101994

**Published:** 2019-06-10

**Authors:** Olga Gruzdeva, Yulia Dyleva, Evgenya Uchasova, Olga Akbasheva, Victoria Karetnikova, Vasiliy Kashtalap, Aleksandr Shilov, Olga Polikutina, Yulia Slepynina, Olga Barbarash

**Affiliations:** 1 Federal State Budgetary Scientific Institution Research Institute for Complex Issues of Cardiovascular Diseases, Kemerovo 650002, Russian Federation; 2 State Budget Educational Institution Higher Professional Education, Siberian State Medical University, Russian Ministry of Health and Social Care Development, Tomsk 634050, Russian Federation

**Keywords:** postinfarction remodelling, growth stimulating factor ST2, NT-proBNP, myocardial infraction

## Abstract

Aim: To assess growth stimulating factor ST2 and N-terminal pro b-type natriuretic peptide (NT-proBNP) levels in the sera of myocardial infraction (MI) patients, and their correlation with the adaptive and maladaptive variants of cardiac remodelling.

Methods: 87 patients (65 male, 22 females; 67±8.36 years) with ST-elevated MI were included in this study, and 67 patients had an adaptive, physiological, while 20 patients had a maladaptive, pathological variant of myocardium remodelling.

Results: On day 1, ST2 and NT-proBNP levels were shown to increase 2.4 and 4.5 folds, respectively, compared with those in the control. ST2 levels in patients with maladaptive remodelling were 1.5-fold higher than those in the adaptive remodelling group. On day 12, a decrease in ST2 levels was observed in both groups. NT-proBNP levels increased 1.8 folds in both groups on day 1, compared with those in the controls. Increased ST2 levels on day 1 after MI were shown to increase the risk of maladaptive remodelling 4.5 folds, while high NT-proBNP levels increased this risk 2.3 times.

Conclusions: ST2 level determination allows us to predict the risk of maladaptive remodelling with a higher sensitivity and specificity than using NT-proBNP levels.

## Introduction

Myocardial infarction (MI) is accompanied by a mechanical deformation of cardiomyocytes, which can be adaptive and maladaptive, leading to chronic heart failure [1]. Currently, myocardial remodelling is considered a complex multistage process, in which the structural and geometric characteristics of left ventricle (LV) are altered and it is manifested as a hypertrophy and dilatation of cardiac chambers with the development of systolic and diastolic dysfunction [2]. A traditionally used indicator of cardiomyocyte stretching and the development and progression of chronic heart failure is N-terminal pro b-type natriuretic peptide (NT-proBNP) [3,4]. However, its application is limited by its biological variations, and its dependence on gender, age and body mass index. NT-proBNP levels in the serum of patients may vary depending on other pathologies as well, such as infections or kidney diseases [4].

Growth-stimulating factor ST2 may represent a more promising marker of early cardiac remodelling, and it is expressed on cardiomyocytes as a response to an increase in biomechanical stress [5]. ST2 is a member of interleukine-1 (IL-1) family receptors [6], and it plays a role in the activation of IL-33, which has antihypertrophic and antifibrotic effects on cardiomyocytes during biomechanical stress. However, an excessive increase in serum ST2 levels is accompanied by the inhibition of the antihypertrophic effects of IL-33 [7]. Therefore, the elucidation of ST2 role and the changes in its levels during the hospitalization of patients suffering from MI may be helpful in predicting the development of LV remodelling and chronic heart failure in postinfarction period.

In this study, we aimed to determine ST2 and NT-proBNP contents in the sera of MI patients during their hospitalization and the potential correlation between the levels of these markers and the development of adaptive or maladaptive variants of cardiac remodelling.

## RESULTS

### Echocardiographic parameters and postinfarction remodelling variant

Patients were divided into two groups according to the variant of postinfarction remodelling. The results of echocardiographic examination are presented in Table 1. Patients with maladaptive remodelling were shown to be characterized by a more severe impairment of systolic and diastolic cardiac function. An increase in the linear dimensions of LV chamber was indicated. Therefore, EDD, ESD, EDV and ESV values were 1.2, 1.3, 1.2 and 1.6 folds, respectively, higher in patients with maladaptive cardiac remodelling, compared with those in patients with adaptive cardiac remodelling.

**Table 1:** Structural and functional myocardial parameters in patients with ST-elevated myocardial infraction, according to the postinfarction remodelling variant.

**Variable**	**Control**, ***n*= 30** Мe (Q1;Q3)	**Patients with adaptive cardiac remodelling** ***n*=67** Мe (Q1;Q3)	**Patients with maladaptive cardiac remodelling** ***n*=20** Мe (Q1;Q3)	***Р***
Ejection fraction (%)	60.5 (60.0;62.0)	52.0 (48.0; 59.0)	43.0 (35.0; 45.0)	Р_1-2_=0.022 Р_2-3_=0.013 Р_1_-_3_=0.016
End-diastolic dimension (сm)	5.2 (5.025;5.3)	5.4 (5.2; 5.8)	6.1 (5.9; 6.4)	Р_1-2_=0.231 Р_2-3_=0.029 P_1_-_3_=0.023
End-systolic dimension (сm)	3.5 (3.4;3.6)	3.9 (3.6; 4.3)	5.0 (4.4; 5.2)	Р_1-2_=0.342 Р_2-3_=0.026 P_1_-_3_=0.021
End-diastolic volume (mL)	130.0 (121.5;135.0)	147.0 (132.5; 169.0)	176.5 (171.0; 209.0)	Р_1-2_=0.187 Р_2-3_=0.031 P_1_-_3_=0.031
End-systolic volume (mL)	52.0 (47.0;54.0)	68.0 (58.0; 83.5)	109.5 (88.0; 124.0)	Р_1-2_=0.025 Р_2-3_=0.032 P_1_-_3_=0.011
Left atrial (cm)	3.95 (3.8;4.0)	4.1 (3.8; 4.3)	4,6 (4.0; 4.7)	Р_1-2_=0.411 Р_2-3_=0.035 P_1_-_3_=0.421
Right atrial (cm)	3.95 (3.8;4.0)	4.0 (3.8; 4.4)	3.9 (3.7; 4.5)	Р_1-2_=0.309 Р_2-3_=0.267 P_1_-_3_=0.341
Right ventricle (cm)	1.8 (1.7;2.0)	1.8 (1.8; 1.8)	1.8 (1.8; 2.0)	Р_1-2_=0.169 Р_2-3_=0.277 P_1_-_3_=0.211
Interventricular septum (сm)	1.0 (1.0;1.1)	1.1 (1.0; 1.2)	1.10 (1.1; 1.2)	Р_1-2_=0.248 Р_2-3_=0.041 P_1_-_3_=0.029
LV posterior wall dimension (cm)	1.0 (0.9;1.0)	1.1 (1.0; 1.2)	1.1 (1.1; 1.3)	Р_1-2_=0.038 Р_2-3_=0.022 P_1_-_3_=0.137
Myocardial weight (g)	224,5 (212,4;244.5)	277.2 (239.9; 289.8)	318.9 (290.5; 378.1)	Р_1-2_=0.111 Р_2-3_=0.078 P_1_-_3_=0.027
Е (cm/с)	68.0 (64.25;70.75)	56.0 (47.0; 66.0)	46.0 (44.0; 54.0)	Р_1-2_=0.255 Р_2-3_=0.032 P_1_-_3_=0.087
А (сm/с)	62.0 (56.25;67.75)	68.5 (57.0; 80.0)	65.0 (50.0; 70.50)	Р_1-2_=0.207 Р_2-3_=0.318 P_1_-_3_=0.133
Е/А	1.12 (1.07;1.22)	0.7 (0.6; 0.9)	0.7 (0.7; 1.4)	Р_1-2_=0.115 Р_2-3_=0.143 P_1_-_3_=0.099
Pulmonary artery pressure (mm Hg)	21.0(19.25;23.0)	25.0 (20.0;28.0)	29.5 (24.0;38.5)	Р_1-2_=0.023 Р_2-3_=0.033 P_1_-_3_=0.251

LV: left ventricular; E: wave of early diastolic filling, A: atrial wave of active filling

LVEF in the maladaptive remodelling group, estimated using the Simpson method, was shown to be 1.2-fold lower than that in the adaptive remodelling group (Table 2) [9]. A decrease in the Е/А ratio was observed in the patients belonging to both remodelling variant groups.

**Table 2:** Baseline clinical and anamnestic characteristics of patients, according to the postinfarction left ventricular remodelling variant Мe (Q1;Q3).

**Variable**	**Patients with adaptive cardiac remodelling** *n*=67	**Patients with maladaptive cardiac remodelling** *n*=20	*P*
Men	49 (73.1%)	16 (80%)	0.54
Arterial hypertension	47 (70.1%)	18 (90%)	0.04
Current smoking	34 (50.7%)	13 (65%)	0.28
Family history of IHD	5 (7.5%)	0 (0%)	0.21
Dyslipidemia	6 (9%)	5 (25%)	0.03
Early postinfarction angina	20 (29.9%)	10 (50%)	0.04
Previous MI	3 (4.5%)	5 (25%)	0.01
Cerebrovascular accident/transient ischemic attack in history	5 (7.5%)	1 (5%)	0.7
Percutaneous coronary intervention history	3 (4.5%)	2 (10%)	0.35
Diabetes/impaired glucose tolerance history	8 (11.9%)	5 (25%)	0.15
**Lesion depth**			
Q-wave MI	47 (70.1%)	18 (90%)	0.33
Non-Q-wave MI	20 (29.9%)	2 (10%)	0.14
**Localization of MI**			
posterior side of the left ventricle	40 (59.7%)	4 (20%)	<0.01
posterior extending to the front side of the right ventricle	10 (14.9%)	2 (10%)	0.11
front side of the left ventricle	17 (25.4%)	14 (70%)	<0.01
**Acute heart failure (Killip)**			
I	58 (86.6%)	9 (45%)	<0.01
II	9 (13.4%)	9 (45%)	<0.01
III	0	1 (5%)	-
IV	0	1 (5%)	-
Cardiac arrhythmias			
Ventricular tachycardia	3 (4.5%)	0	0.36
Atrial fibrillation	3 (4.5%)	2 (10%)	0.37
**Third-degree atrioventricular block**	2 (3%)	1 (5%)	0.29
Early post-infarction angina	3 (4.5%)	2 (10%)	0.35
Recurrent MI	2 (3%)	1 (5%)	0,66
Heart failure	2 (3%)	0	-
coronary artery bypass grafting	0	2 (10%)	-
Death	1 (1.5%)	0	-
**Treatment strategy/drug groups**			
β-blockers	66 (98.5%)	20 (100%)	0.58
Angiotensin-converting-enzyme inhibitor	54 (80.6%)	15 (75%)	0.59
Calcium channel blocker	46 (68.7%)	14 (70%)	0.91
Loop diuretics	20 (29.9%)	14 (70%)	0.01
Thiazide diuretics	2 (3%)	3 (15%)	0.04
Spironolactone	24 (35.8%)	14 (70%)	0.01
Nitrates	8 (11.9%)	7 (35%)	0.02
Aspirin	67 (100)	20 (100)	-
Heparin	35 (52.2%)	15 (75%)	0.07
Clopidogrel	65 (97%)	20 (100%)	0.99
Statins	47 (70.1%)	11 (55%)	0.21
Thrombolytic therapy	8 (11.9%)	3 (15%)	0.72
Percutaneous coronary intervention	64 (95.5%)	20 (100%)	0.98

IHD: ischemic heart disease; MI: myocardial infraction.

According to the patients’ medical records, coronary artery disease (CAD) risk factors were more frequently observed in patients belonging to the maladaptive remodelling group, than in the adaptive remodelling group, such as AH, observed in 90% of patients in this group, while in 50% of patients, clinical signs of angina pectoris were noticeable prior to the MI development. In 25% of patients with maladaptive remodelling, MI had been previously diagnosed (Table 2).

Q-wave MI and anterior localization were observed more frequently in patients with the pathological variant of remodelling. In contrast to this, non-Q-wave MI and posterior localization were more frequently observed in patients with adaptive cardiac remodelling. Adaptive remodelling was shown to correlate significantly with the presence of acute heart failure Killip class I, while patients in the maladaptive remodelling group were shown to have acute heart failure of Killip classes II-IV.

The scope of the applied therapy did not differ between the groups, but the patients with maladaptive cardiac remodelling were shown to be prescribed loop, thiazide, potassium-sparing diuretics and nitrates more often.

### Cardiac remodelling markers

ST2 and NT-proBNP levels in the sera of patients belonging to the adaptive and maladaptive cardiac remodelling group, at day 1 after the MI, were shown to be higher than those in the control group. ST2 levels were 2.3- and 1.4-fold higher, respectively, while those of the NT-proBNP were 3.5- and 5.3-fold higher, respectively.

On day 12 after the MI, significant changes were observed only in the ST2 levels, which decreased in the adaptive and maladaptive cardiac remodelling groups, 2.5 and 3.8 folds, respectively.

**Figure 1:**
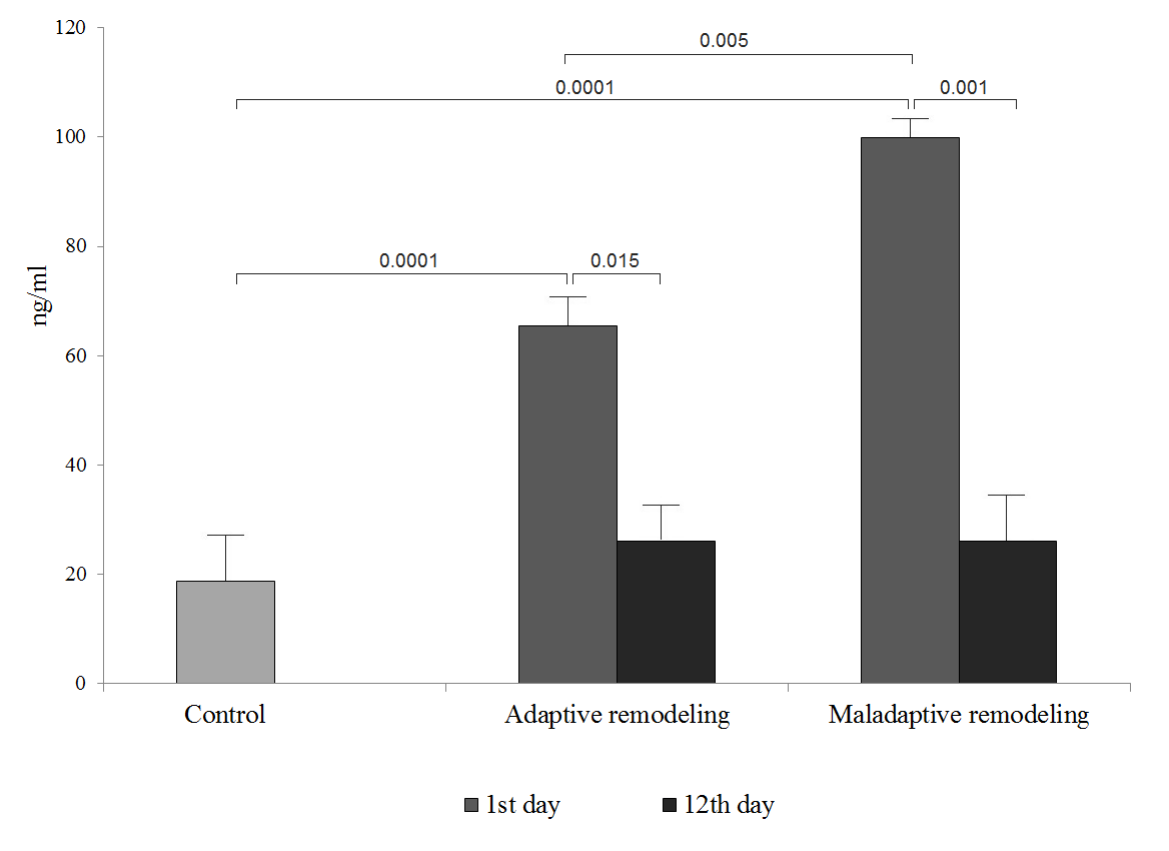
**Stimulating growth factor ST2 levels in the sera of patients with either adaptive or maladaptive cardiac remodelling variants.**

**Figure 2:**
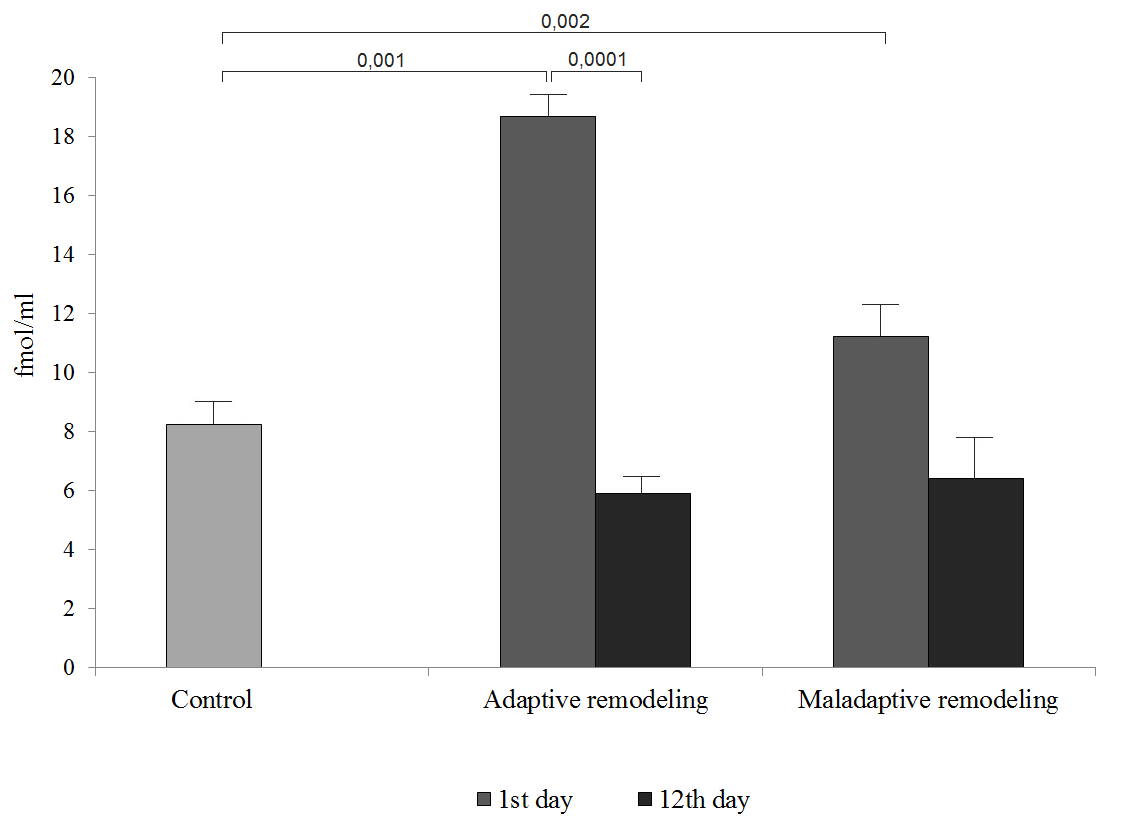
**N-terminal pro b-type natriuretic peptide levels in the sera of patients with either adaptive or maladaptive cardiac remodelling variants.**

We demonstrated that the increased levels of ST2 on day 1 after the MI were associated with the development of the pathological variant of postinfarction remodelling, and its levels were 1.5 folds increased in comparison with those in the adaptive remodelling group. However, NT-proBNP levels were similar in both groups during the hospitalization period (Fig. 1, 2).

We analysed the relationships between ST2 levels and LV functional characteristics. We demonstrated an inverse correlation between the ST2 and LVEF (r=-0.545, *P*=0.001) and direct correlation between medium strength and EDV (r=0.510, *P*=0.003) or ESV (r=0.463, *P*=0.001). However, NT-proBNP levels were shown to be inversely correlated with EDV volume alone (r=-0.514, *P*=0.010). Moreover, we were interested in the correlation analysis between troponin T (the classic marker of cardiomyocyte damage) and sST2 concentrations during the MI hospitalization period. The results of the correlation analyses indicated a direct correlation between the levels of sST2 and troponin T in both groups at the time of admission (R = 0.65, P = 0.002) (Table 3).

**Table 3:** Correlation of N-terminal pro b-type natriuretic peptide, stimulating growth factor ST2 levels and echocardiography parameters.

**Variable**		**Ejection fraction (%)**	**End-diastolic dimension (сm)**	**End-systolic dimension (сm)**
ST2, ng/ml	R	-0.545	0.510	0.463
	P	0.001	0.003	0.000
NT-proBNP, fmole/ml	R	-	-0.514	-
	P	-	0.010	-

**Figure 3:**
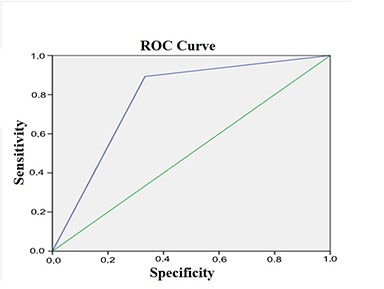
**ROC curve analysis associations of serum level ST2 (1st day after MI) with the risk of non-adaptive left ventricular remodeling.**

**Figure 4:**
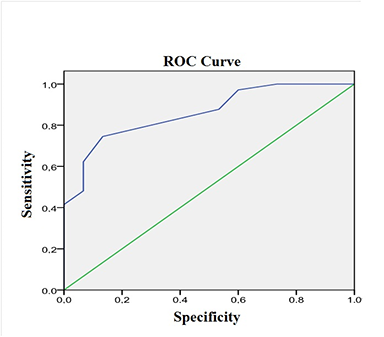
**ROC curve analysis association of serum NT-proBNP with the risk of non-adaptive left ventricular remodelling.**

Using the logistic regression analysis, we determined that ST2 levels measured on day 1 after the MI are associated with the development of maladaptive remodelling of LV (OR=4.5, 95% CI=2.0-10.1, *P*=0.011, AUG=0.81; sensitivity, 78.7%; specificity, 69.4%) (Fig.3), while the levels of NT-proBNP were less significantly associated with the risk of maladaptive remodelling (OR=2.3, 95% CI=2.0-2.01, *P*=0.032, AUG=0.68; sensitivity, 69.5%; specificity, 45.9%) (Fig.4).

## DISCUSSION

The adaptive or physiological variant of cardiac remodelling assumes the absence of the signs of heart failure and minor changes in LV structure. The presence of these symptoms and a progressive heart failure determines the maladaptive or pathological variant of remodelling, which is crucial for the formation and progression of chronic heart failure in patients after the MI [1,2]. Here, we observed that almost a fifth of all patients with STEMI in the postinfarction period showed signs of maladaptive cardiac remodelling.

Maladaptive sequelae after MI often contribute to the unfavourable course of the disease and the mortality of the patients. Despite the significant progress in treatment development, the prognosis is still unfavourable for these patients [9,10].

Here, we determined that the cardiac rhythm disorders, such as ventricular fibrillation, third-degree atrioventricular nodal block, early postinfarction angina and the recurrent MI were more often observed among the patients with maladaptive cardiac remodelling.

In response to the hemodynamic stress and during LV postinfarction remodelling, a number of humoral factors are secreted, including NT-proBNP, an indicator of myocardial stretching [4,7,11], which is considered an adaptive response to injury. NT-proBNP renin-angiotensin-aldosterone system antagonist, its levels increase during natriuresis and diuresis, and it induces vasodilation, reduces heart preload and afterload and blood pressure, and has anti-ischemic effects [12–14]. This molecule is used as a biological marker, which is secreted before troponin can be detected in blood, and its levels reflect the severity of structural and functional impairments of the myocardium [3,4]. NT-proBNP levels were shown to decrease significantly in the patients with adaptive cardiac remodelling, while no significant changes were observed in the maladaptive remodelling group.

Several studies reported a statistically significant association between NT-proBNP levels and the impaired LV structure and function during postinfarction remodelling, specifically with LV dilatation and systolic dysfunction, which are considered the most unfavourable factors for the development and progression of heart failure [15,16]. However, in this study, only an inverse correlation between the NT-proBNP levels and EDV was observed.

Furthermore, the alterations in the levels of ST2 were more pronounced, since the levels of this molecule increased on day 1 after the MI and decreased by day 12. The increase in the levels of ST2 are associated with the increase in the levels of its synthesis in cardiomyocytes and fibroblasts due to biomechanical stress [7,17,18]. Moreover, ST2 levels were shown to be associated with the functional activity of LV, necrotic zone size. Additionally, this molecule was shown to be a marker of postinfarction remodelling: in patients with heart failure, the disturbance of ST2 signal transmission leads to the gradual remodelling of myocardium [19]. Previous studies showed that ST2 levels are associated with the severity of heart failure, regardless of the risk associated with the increase in NT-proBNP levels and the impact of other risk factors [20–23].

Here, we showed that ST2 levels on day 1 after the MI are significantly higher in the patients with maladaptive cardiac remodelling, compared with those in the adaptive remodelling group. The increase in ST2 levels during the maladaptive remodelling may be connected with the increase in the levels of soluble form of this marker during the cardiomyocyte injury. Additionally, following the MI, the activation of humoral and cellular immune response, which is necessary for cicatrization of the necrotic zone, contributes to the increase in ST2 levels [9]. Furthermore, increased levels of this marker can be explained by considerable hemodynamic decompensation and the induction of inflammation during ischemia/reperfusion [8].

The results demonstrating that by day 12 after the MI, the levels of ST2 decreased, and were similar to the levels determined in the controls. These results are consistent with the previously reported results, obtained using an experimental mouse model of MI, by ligating coronary artery. It was shown that the maximal induction of ST2 transcription in cardiomyocytes occurs 2 h after the MI, lasts for 9 h and decreases after 15 h [24].

Maladaptive cardiac remodelling is characterized by a progressive increase in the volume and wall thickness of LV cavity. Structural changes are accompanied by a decrease in myocardial contractility and diastolic function [18]. Therefore, the results of this study demonstrated a correlation between ST2 levels and EF, EDV and ESV. Furthermore, we observed a decrease in the E/А ratio, indicating impaired LV myocardium relaxation and diastolic dysfunction. Rhythm disturbances were more often observed in patients with the pathological variant of postinfarction remodelling. The obtained data are consistent with the results obtained in the large-scale studies including patients with acute MI, which revealed the correlations with LVEF in early time-points following the MI [15,18].

## CONCLUSION

We observed maladaptive cardiac remodelling in 23% of 87 patients included in this study. ST2 levels were shown to change significantly during the hospitalization period after the MI, but they return to the levels of control by day 12 after the MI. Maladaptive remodelling was shown to be associated with increased levels of this marker, in contrast to that of the NT-proBNP, which did not differ between the analysed groups. ST2 levels on day 1 after the MI may be a useful marker for the prediction of maladaptive remodelling risk, and this marker was shown to have a higher sensitivity and specificity than NT-proBNP.

## METHODS

This study and all experimental protocols used were approved by the Ethics Committee of the Federal State Budgetary Institution, Research Institute for Complex Issues of Cardiovascular Diseases (protocol No. 10 from 10.06.2015), and was developed in accordance with the Declaration of Helsinki (“Ethical principles for medical research involving human subjects”, amended in 2000) and “Rules for clinical practice in the Russian Federation”, approved by the Ministry of Health of the Russian Federation on 19 June 2003. Written informed consent was obtained from all patients prior to their enrolment.

### Study participants

The inclusion criterion was the ST-elevated MI (STEMI) within 24 h prior to the hospital admission, with no age restrictions. The exclusion criteria were: MI requiring complicated percutaneous coronary intervention (PCI) or coronary artery bypass grafting, terminal kidney failure (glomerular filtration rate<15 mL/min), a history of diabetes mellitus and diabetic comas, a known cancer history, the presence of other diseases that may significantly reduce life expectancy, including systemic diseases of connective tissue and other potential inducers of cardiac remodelling, such as malignant arterial hypertension (AH), valvular diseases, cardiomyopathy, arrhythmias and others.

The diagnosis of STEMI was established according to the National Guidelines 2007 of the Russian Society of Cardiology, which includes clinical data indicating angina lasting for 20 min or more, electrocardiographic findings indicating ST-segment elevation by 0.1 mW in two or more contiguous leads or new-onset complete left bundle branch block in ECG and an increase in the level of troponin T greater than 0.1 ng/mL. At least two of these criteria had to be satisfied, while the increase in the levels of biochemical markers of myocardial necrosis was a necessary condition. MI severity class was evaluated using the Killip classification (1967) [25].

### Study population

Eighty-seven patients (65 male, 22 female) with STEMI, admitted to the hospital within 24 h from the moment of clinical symptom manifestation, were enrolled in the study during one calendar year (2015). Clinical and anamnestic characteristics are presented in Table 4.

**Table 4:** Baseline clinical and anamnestic characteristics of patients enrolled in this study Мe (Q1;Q3).

**Variable**	*n*=87	%
Men	65	75
Arterial hypertension	65	75
Current smoking	47	54
Family history of IHD	22	25
Dyslipidemia	26	30
Early post-infarction angina	30	34
Previous MI	8	9
Cerebrovascular accident/transient ischemic attack in history	6	7
**Lesion depth**		
Q-wave MI	65	75
Non-Q-wave MI	22	25
**Localization of MI**		
posterior	44	51
posterior extending to the front side of the right ventricle	12	14
front side of the left ventricle	31	36
**Acute heart failure (Killip)**		
I II III IV	67 18 1 1	77 21 1 1
Ventricular tachycardia	3	3
Atrial fibrillation	5	6
Atrioventricular block III	3	3
Early post-infarction angina, n (%)	5	6
Recurrent MI	3	3
**Comorbidities**		
Chronic bronchitis	19	22
Peptic ulcer disease in remission	17	20
Chronic pyelonephritis	20	23
**Treatment strategy/group of drugs**		
β-blockers	82	94
angiotensin-converting-enzyme inhibitor	69	79
Calcium channel blocker	60	69
Loop diuretics	34	39
Thiazide diuretics	5	6
Spironolactone	38	44
Nitrates	15	17
Aspirin	87	100
Heparin	50	57
Clopidogrel	75	96
Statins	68	77.3
Thrombolytic therapy	11	13
Percutaneous coronary intervention	84	97

IHD: ischemic heart disease; MI: myocardial infraction.

Patients with no contraindications during the hospital stay were prescribed combined coronary active anti-thrombotic therapy according to the standard practice (Table 1). Statins were administered to 58 (67%) patients. As a reperfusion therapy, a primary PCI in the culprit artery was implemented in 97% of the patients, and 13% of patients received systemic thrombolysis with 1.5×10^6^ IU of streptokinase.

### Echocardiography

On day 10-12, all patients underwent echocardiography using ALOKA ProSound α-10 Model SSD-α10 (ALOKA CO, LTD. Tokyo, Japan) in M- and B-modes, in pulsed wave and constant wave Doppler modes, colour flow Doppler echocardiography, in tissue mode, Doppler and in colour M-mode, Doppler echocardiography, using ultrasound array sensor at 2-4 MHz. The examination was performed in standard positions. The following indicators of structural and functional state of cardiovascular system were evaluated: ejection fraction (EF), left atrial (LA) dimension, LV end-diastolic dimension (EDD), LV end-systolic dimension (ESD), LV end-diastolic volume (EDV), LV end-systolic volume (ESV), interventricular septum (IVS) dimension and LV posterior wall dimension (LVPW). Relative wall thickness index was calculated as well, according to the following formula: RWT=IVST+LVPWT)/LV EDD). Patients were divided into two groups, based on the presence of adaptive or maladaptive remodelling. According to the National Guidelines of the Russian Society of Cardiology and the Society of Heart Failure Specialists on diagnosis and treatment of chronic heart failure, the remodelling was considered adaptive if: (a) LVEF≥45% and/or LV EDD<5.5 cm; (b) RWT=0.3–0.45 cm; (c) diastolic dysfunction in the form of (IVST+LVPWT)=1.3–2.0 cm, and/or LVPWT>1.2 and/or hypertrophic type of transmitral Doppler flow spectrum (TMDF), Е/А (wave of early diastolic filling/atrial wave of active filling)=1.1–2.0. Maladaptive remodelling was characterized by the following: (a) LVEF≤45% and/or LV EDD>5.5 cm; (b) RWT≤0.3 cm; (c) diastolic dysfunction in the form of (IVST+LVPWT)>2.0 cm and/or Е/А=1.1-2.0. Adaptive remodelling group included 67 patients, while the maladaptive remodelling group included 20 patients. The control group consisted of 30 patients without cardiovascular diseases who were compatible in gender and age with the patients of the main group.

### Laboratory assays

Serum was separated from venous blood by centrifugation at 3,000 ×*g* for 20 min and stored at -70°C. ST2 levels were measured using Presage ST2 assay (Critical Diagnostics, San Diego, CA, USA). This assay has a within-run coefficient of variation (CV)<6.5% and total CV<9.1% at a mean concentration of 16.9 ng/mL. We determined NT-proBNP levels with the Biomedica kit (Bratislava, Slovakia). The intra-assay CVs were 5% and 8% at a mean concentration of 13 fmol/mL. Troponin T levels were measured with Roche CARDIAC (Roche Diagnostics, Mannheim, Germany). All Roche assays were performed with the use of the Elecsys 2010 system (Roche Diagnostics): Troponin T (fourth generation) with a limit of detection of 0.01 ng/mL, a 99th-percentile cutoff point of less than 0.01 ng/mL, and a CV of less than 10% at 0.035 ng/mL.

### Statistical analysis

Statistical analyses of data obtained in this study was performed using software tool STATISTICA 6.1 (StatSoft, Tulsa, OK, USA) and SPSS 10.0 for Windows (SPSS Inc., Chicago, IL, USA). The results are presented as median and the first and third quartiles (Q1 and Q3). We used the non-parametric Mann-Whitney-Wilcoxon tests for the analysis of quantitative data that was not normally distributed. Spearman correlation analysis was used to determine the relationship between the variables. The analysis of the frequency ratio differences between two independent groups was performed using the Fisher's exact test with two-sided confidence interval. *P* values lower than 0.05 were considered statistically significant. The identification of the most informative indicators for the estimation of postinfarction remodelling with the determination of odds ratio (OR) and 95% confidence interval (CI) was performed by using stepwise logistic regression analysis and defining the area under receiver operating curve (AUC).

### Ethical approval

The study protocol was approved by the local ethics committee of the Federal State Budgetary Scientific Institution Research Institute for Complex Issues of Cardiovascular Diseases.
